# Pan-genomic insights into LTP gene family evolution across diploid cotton species

**DOI:** 10.3389/fpls.2025.1691339

**Published:** 2025-12-22

**Authors:** Yanghan Lu, Lishuang Guo, Zhengya Wei, Yujun Li, Yue Zhang, Juyun Zheng, Baohua Wang, Zhonghua Zhou, Haodong Chen

**Affiliations:** 1 College of Agronomy, Hunan Agricultural University, Changsha, Hunan, China; 2 Institute of Cotton and Sericulture, Hunan Academy of Agricultural Sciences, Changsha, Hunan, China; 3 Yuelushan Laboratory, Changsha, Hunan, China; 4 Cotton Research Institute of Xinjiang Uygur Autonomous Region Academy of Agricultural Sciences, Urumqi, Xinjiang, China; 5 School of Life Sciences, Nantong University, Nantong, Jiangsu, China

**Keywords:** *Gossypium*, lipid transfer protein (LTP), pan-genome analysis, gene family evolution, structural variation, selection pressure, transcriptional regulation, fiber development

## Abstract

**Introduction:**

Lipid-transfer proteins (LTPs) are a class of small, alkaline proteins that bind and transport various lipid molecules, including fatty acids, phospholipids, glycolipids, and steroids, between phospholipid bilayers. They play crucial roles in signal transduction, stress tolerance, and plant growth and development.

**Methods:**

In this study, based on pan-genomic data, we identified 107 LTP family members across nine diploid cotton species, comprising 45 core, 43 variable, and 19 specific genes. Synteny and selection pressure analyses clarified the evolutionary relationships among these genes, while structural variation analyses revealed that although structural variants altered gene structures, domains, and cis-acting elements, they did not significantly affect gene expression.

**Results:**

Expression profiling further demonstrated that LTP genes exhibited distinct spatiotemporal expression patterns in cotton ovules and roots at different developmental stages.

**Discussion:**

Overall, these findings highlight both conserved and divergent evolutionary patterns of the LTP family among diploid cotton species, providing new insights into their functional diversification, adaptive evolution, and potential involvement in cotton fiber development and stress responses.

## 1 Introduction

Plant lipid transfer proteins (LTPs) are a class of small, alkaline proteins. Their tunnel-like hydrophobic chamber, which allows them to effectively bind and transmit different lipid complexes between phospholipid bilayers, is the most characteristic structural feature of LTPs. These lipid complexes include fatty acids, phospholipids, glycolipids, and steroids (Edstam et al., 2011). LTPs possess a compact three-dimensional structure, which is stabilized and maintained by four conserved disulfide bonds. An octa-cysteine motif (8CM) is responsible for the formation of these four disulfide bonds. C-Xn-C-Xn-CC-Xn-CXC-Xn-C-Xn-C is the generic form of the 8CM motif, where C stands for a cysteine residue, X for any amino acid, and n for any number of amino acids (Gincel et al., 1994). Nearly all nsLTPs contain an N-terminal signal peptide that directs the protein to its specific subcellular location (Liu et al., 2015). LTPs are classified on the basis of the following aspects: the distance between cysteine residues in the 8CM region, peptide sequence similarity, and evolutionarily conserved intron positions. On the basis of these criteria, LTPs are categorized into five primary categories: LTP-1, LTP-2, LTP-c, LTP-d, and LTP-g, as well as five secondary categories: LTP-e, LTP-f, LTP-h, LTP-j, and LTP-k (Edqvist et al., 2018; Missaoui et al., 2022). Additionally, the classification of LTPs is also related to posttranslational modifications. For instance, LTPs with GPI anchors are classified as the LTPg type. Such posttranslational modifications may significantly impact the localization of LTPs in cells and their functions (Edstam et al., 2011).

As research in this field has advanced, the LTP family is known to be extensive and to exhibit considerable structural diversity across various plant species. The expression of LTPs is induced by various environmental stresses, such as drought, cold, salt-related stress, and pathogens from bacteria and fungi (Akhiyarova et al., 2020; Brotman et al., 2012). In response to nonbiotic stress, LTP proteins have been shown to participate in the formation of the plant epidermal barrier by transporting cutin monomers and wax precursors to regulate cuticle development, thereby reducing water loss and improving environmental adaptability (Edqvist et al., 2018). The overexpression of *NtLTP4* in tobacco plants reduces water loss by lowering transpiration rates and maintaining ion homeostasis under drought and high-salt stress by increasing their ROS-scavenging capacity. These combined benefits improve drought tolerance and salt stress resistance in tobacco plants (Xu et al., 2018). By controlling the expression of genes linked to oxidative stress, the rice *OsLTPL159* gene increases plant resistance to low temperatures (Zhao et al., 2020). Studies have shown that LTPs can inhibit the growth of pathogenic microorganisms, contributing to resistance against bacterial and fungal infections. *In vitro*, for example, the overexpression of *SlLTPg1* in tomatoes enhances their resistance to late blight and results in direct antibacterial activity against Fusarium oxysporum *in vitro* (Liu et al., 2024). During seed development, LTPs facilitate lipid accumulation and seed germination through the transport of lipids and proteins. For example, loss of function of *OsLTPL36* in rice leads to a decrease in lipid content, resulting in insufficient energy and nutrients required for seed germination and seedling growth, thereby impairing these processes (Wang et al., 2015). Additionally, LTPs play crucial roles in anther development. Mutations in genes related to lipid metabolism can result in genetic male sterility (GMS), manifested as abnormalities in anther development and abnormal suppression of anther length (Wan et al., 2020). Male sterility results from the disruption of cuticle and pollen wall production caused by loss of function of LTP genes that are unique to or strongly expressed in anthers (Fang et al., 2023).

In the field of cotton research, significant progress has been made in the study of LTPs. The cotton *GhLTP4* gene plays a crucial role in mediating responses to abiotic stress, particularly drought conditions. Research has shown that, through mechanisms such as reshaping the lipid profile, regulating ABA signal transduction, and improving the TCA cycle, plants overexpressing GhLTP4 increase the water retention capacity of the cuticle, maintain membrane stability, and significantly improve cotton drought tolerance (Zhang et al., 2022). In response to pathogens and pests, the *GhnsLTPsA10* gene exhibits tissue-specific expression patterns. The upregulation of this gene in the roots enhances the resistance of cotton to fungal pathogens, whereas its downregulation in the leaves improves resistance to insects. This pattern effectively coordinates the resistance of cotton to both fungal pathogens and insects (Chen et al., 2021a). The continued synthesis and transport of lipids and proteins are essential for the expansion of vesicles and plasma membranes during cotton development, with LTPs playing a central role in this process (Gou et al., 2007). Earlier studies have shown that genes such as *LTP3*, *LTP6*, and *LTP12* are especially expressed during the extension phase of cotton fiber formation, where they significantly influence fiber cell growth, elongation, and cell wall synthesis (Liu et al., 2000; Ma et al., 1997). Further studies revealed that *GhLTPXIs* are highly expressed during fiber elongation in long-fiber cotton varieties. When ectopically expressed in *Arabidopsis*, *GhLTPXIs* significantly increase trichome length, suggesting their role in promoting fiber elongation (Meng et al., 2018). Furthermore, phosphatidylinositol monophosphates (PtdInsPs) are selectively bound and transported by GhLTPG1, a cotton GPI-anchored lipid transfer protein. Cotton fibers become shorter when the GhLTPG1 gene is silenced because elongated fibers have less PtdInsP (Deng et al., 2016). Although some progress has been made in the study of LTPs in cotton, many limitations remain. On the one hand, current research on LTP function in cotton has focused primarily on a few genes, and comprehensive knowledge of the entire *LTP* gene family remains lacking. On the other hand, the complexity and diversity of the cotton genome make it difficult to distinguish differences accurately between genomes via traditional gene family identification methods, hindering a more thorough comprehension of the function and evolution of the *LTP* gene family.

With the continuous improvement in cotton genomic data, pan-genome-based gene family analysis offers a new opportunity to comprehensively reveal the functions and evolution of the *LTP* gene family. Compared with a single reference genome, pangenomic analysis can complement missing gene family members from the reference genome, revealing additional variation and diversity in gene families (Sun et al., 2022). Diploid cotton, as the ancestor of tetraploid cotton, is highly important for understanding the formation and evolutionary mechanisms of tetraploid cotton through in-depth studies of its genome structure and function (Chen et al., 2017; Chen et al., 2017). However, there is a lack of current research on the mechanisms of genetic regulation of fiber quality in diploid cotton species, leading to unclear genetic commonalities in domestication and breeding selection, which limits the use of superior genetic variation in interspecific breeding.

In this study, the *LTP* gene family was systematically identified and analyzed on the basis of the pan-genomes of nine high-quality diploid cotton species. The evolutionary patterns of the LTP gene family in diploid cotton species were fully revealed by identifying the LTP family, studying its evolution in these species, and examining the impacts of structural variation. In parallel, the differential expression of *LTP* genes in cotton fibers at different stages was analyzed to identify *LTP* genes with potential functions that may provide new genetic resources and a theoretical basis for cotton fiber quality improvement and stress tolerance breeding. In addition, examining interspecies variations among diploid cotton species is essential for understanding how evolutionary divergence has shaped the LTP gene family and contributed to species-specific adaptation. The results of these studies will not only deepen the understanding of the mechanisms of the *LTP* gene family in plant growth, development, and stress responses but also provide important references and guidance for cotton genetic breeding and molecular design breeding.

## 2 Materials and methods

### 2.1 Identification of the LTP gene family in diploid cotton species

The genomic data used in this study to identify LTP family members were derived from Wang et al (Wang et al., 2022). A total of nine cotton species were included in the genomic data, specifically, *Gossypium herbaceum* (A1), *Gossypium arboreum* (A2), *Gossypium anomalum* (B1), *Gossypium sturtianum* (C1), *Gossypium raimondii* (D5), *Gossypium stocksii* (E1), *Gossypium longicalyx* (F1), *Gossypium bickii* (G1), and *Gossypium rotundifolium* (K2). To identify LTP family members accurately, the typical structural features of *LTP*, which include four α-helices, one β-fold, four pairs of disulfide bonds (Cys1-5, Cys2-6, Cys3-7, and Cys4-8), and an N-terminal signal peptide, were identified. These characteristics were used to retrieve hidden Markov models (HMMs) of the LTP family’s structural domains (PF14368, PF00234) from the Pfam database (https://www.ebi.ac.uk/interpro/). Subsequently, HMMER version 3.3.2 was used to search the above cotton genome datasets with an E-value threshold of <1E−5 and an alignment coverage greater than 90% to identify potential LTP domains and obtain candidate *LTP* genes. In addition, to identify atypical genes, the alignment coverage criterion was slightly relaxed, and genes with coverage greater than 80% were also included for further analysis. To ensure the accuracy of the identification results, both sets of candidate LTP genes were further validated using the SMART (https://smart.embl.de/), NCBI CDD (https://www.ncbi.nlm.nih.gov/Structure/cdd/cdd.shtml), and PFAM (https://www.ebi.ac.uk/interpro/) databases to confirm whether they truly contained LTP domains, and incorrectly annotated genes were removed to reduce the false-positive rate.

### 2.2 Phylogenetic tree and presence/absence variant analysis of the *LTP* gene family in diploid cotton species

Using bioinformatics tools like MUSCLE (https://www.drive5.com/muscle/), Trimal (http://trimal.cgenomics.org/), and IQ-TREE2 (http://www.iqtree.org/), a phylogenetic tree containing 23 Arabidopsis LTP family proteins and 107 identified cotton *LTP* family proteins was created for this study. Evolview 3.0 (https://www.evolgenius.info/evolview/) software was subsequently used to visualize and map the phylogenetic tree. Additionally, in the *LTP* gene family variation analysis, data on *LTP* presence/absence variants (PAVs) from (Wang et al., 2022) were referenced, and R script 4.0.3 was used to create presence/absence heatmaps of the PAVs of *LTP* genes in the nine diploid cotton species.

### 2.3 Ka/Ks calculation

Genomic data from nine diploid cotton species were obtained from Wang et al (Wang et al., 2022), and protein sequences of *LTP* genes, along with their corresponding coding sequences (CDSs), were screened. The *LTP* gene sequences were then analyzed, and Ka/Ks values for each *LTP* gene were calculated via KaKs_Calculator software to assess selection pressure. The distribution characteristics of the Ka/Ks values were visualized via an in-house Perl script to generate a Ridgeline plot. Furthermore, to further explore the positive selection of *LTP* genes, Rscript 4.0.3 software was used to generate heatmaps of the proportions of *LTP*-based genes with Ka/Ks values greater than 1 in different cotton species.

### 2.4 Expression analysis of *LTP* genes overlapping with SV

In this study, information on the location of structural variants (SVs) in each variety, along with gene expression data from nine materials, was obtained from the study by Wang et al. The overlap between structural variations (SVs) and lipid transporter protein genes (LTPs) in each cotton variety was determined using a custom Perl script. The matching gene expression data for that cotton species were categorized as related to the SVs if an LTP gene coincided with one. In the absence of overlap, the expression data were considered not to contain SVs. The relationship between the expression levels of genes and the existence of overlapping SVs was then examined using the Pearson correlation coefficient. LTP genes with *p < 0.05* and |r| > 0.3 were determined to be those whose expression levels were significantly changed by SVs based on the computation findings. Additionally, the Wilcoxon test was applied to detect and analyze differences between atypical and typical *LTP* genes.

### 2.5 Analysis of *LTP* gene structure

Gene annotation files in the generalized feature format (GFF) were retrieved from the CottonGen database CottonGen (https://www.cottongen.org/). The MEME Suite v5.4.1 tool (https://meme‑suite.org/meme/) was employed to analyze motifs in cotton LTP protein sequences, with the motif number fixed at 10.The gene structure of cotton *LTP* was then mapped using TBtools (v1.098761) software by merging the downloaded GFF genome annotation data with the previously described base-sequencing result files.

### 2.6 *Cis*-acting elements of promoters

Sample A1, which exhibited the highest degree of overlap with structural variants (SVs) in the above data, was screened, and its promoter sequence was extracted. Simultaneously, the 2000 bp promoter sequence of the corresponding gene in reference genome A2 was obtained. The extracted promoter sequence of sample A1, along with the 2000 bp promoter sequence of reference genome A2, was submitted to the PlantCARE database (http://bioinformatics.psb.ugent.be/webtools/plantcare/html/) for *cis*-acting element analysis (Lescot et al., 2002). Upon completion of the analysis, the resulting *cis*-acting elements were visualized via TBtools software (Chen et al., 2023).

### 2.7 RNA-seq data analysis

In this study, the transcriptome data of three genomes, B1, C1, and K2, detected by our team, were analyzed, along with data from other diploid cotton species provided by Wang et al (Wang et al., 2022). Furthermore, the expression values of the A2 genome were additionally extracted from the **Supplementary Table** in the study of Hu et al (Hu et al., 2022). The expression of LTP family members in diploid cotton species was subsequently analyzed across different developmental periods in the roots and ovules. For the analysis, Rscript 4.0.3 software was used to generate heatmaps on the basis of log2(RPKM+1) values, which were used to visualize the gene expression patterns. Additionally, several common transcription factors were screened from the table, and their expression data were extracted. In-house-written Perl scripts were then used to analyze and visualize the coexpression of differentially expressed family genes and transcription factors.

## 3 Results

### 3.1 Identification of pangenomic LTP in diploid cotton

**Figure 1:**
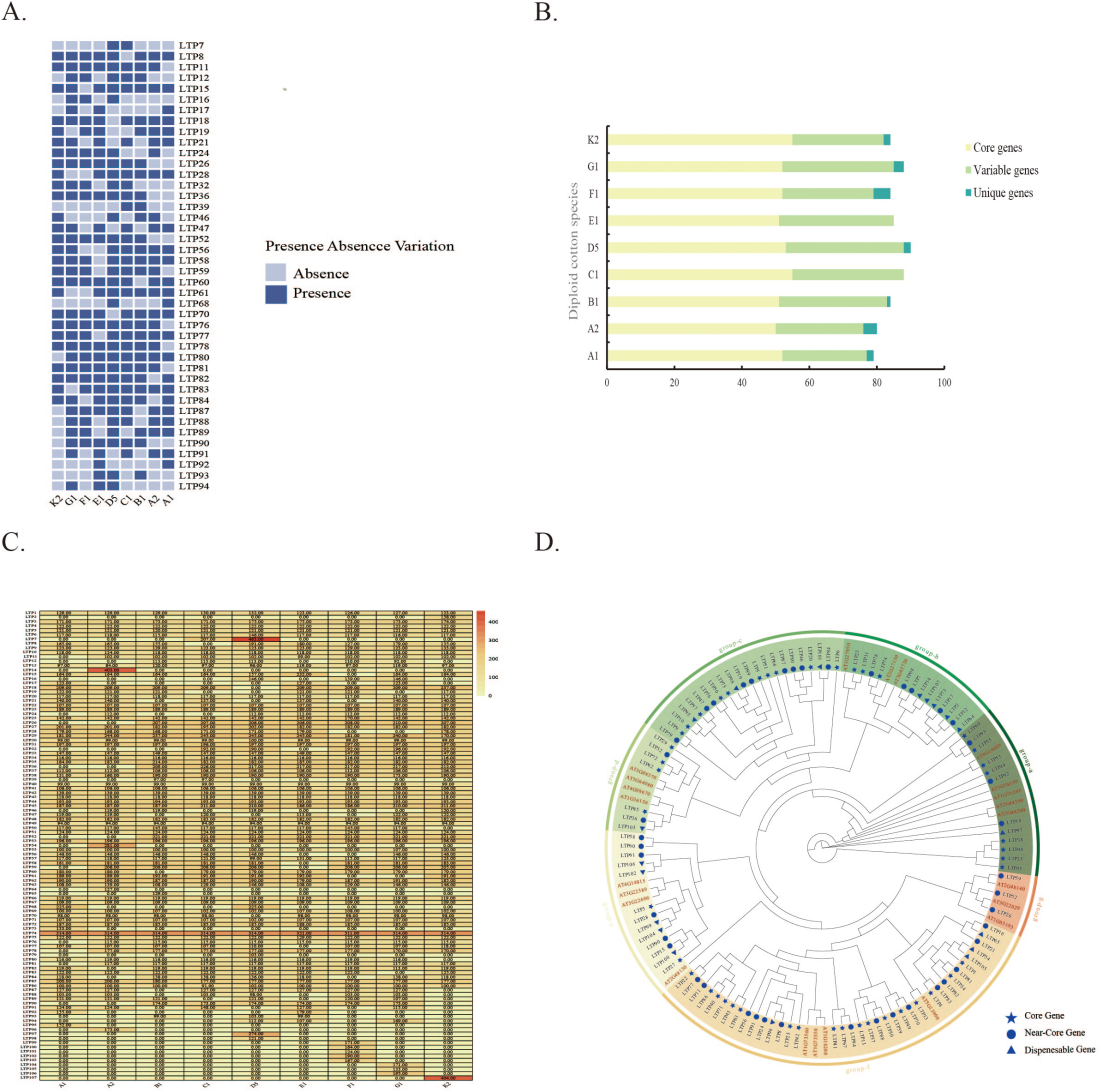
Phylogenetic investigation and identification of LTPs in the diploid cotton pan-genome. **(A)** Heatmap depicting the presence of 42 LTPs with deletions, excluding core and exclusive genes, in nine diploid cotton species. **(B)** Number of LTPs in core genes, variable genes, and exclusive genes across nine samples. **(C)** Heatmap showing the length distribution of LTP proteins. **(D)** Phylogenetic tree of LTPs in Arabidopsis and diploid cotton species.

In this study, *LTP* gene identification was systematically conducted via pangenomic data from nine diploid cotton species, resulting in the identification of 107 *LTPs* (**Supplementary Table 1**). PAV, an important type of gene structural variation, reflects differences in the distribution of genes across genomes. In this study, the PAV analysis strategy was applied within the framework of pangenomic analysis to identify *LTP* gene deletions across different genomes. Analysis of *LTP* gene deletions, excluding endemic genes, across the nine cotton species presented in **Figure 1A** revealed that the pan-genome of the genus Cotton contains a core gene family, a variable gene family, and a specific gene family, with 45 core genes, 43 variable genes, and 19 specific genes. Additionally, statistical analysis of the gene type distribution across the nine cotton species revealed that core genes constituted 61.9% of all *LTP* genes, variable genes constituted 35.6%, and specific genes constituted 2.5%. Notably, specific genes, C1 and E1, were not detected in the two cotton species (**Figure 1B**). Core gene families were prevalent across all analyzed genomes, whereas specific gene families appeared only in some genomes. This diversity in gene family distribution reflects the complexity and adaptive nature of the Cotton genus genomes.

To thoroughly investigate the protein length characteristics of *LTP* family members across different cotton samples, the relevant data were further examined in this study (**Figure 1C**). The findings showed that among the analyzed LTP family members, 13 genes—*LTP4*, *LTP20*, *LTP22*, *LTP34*, *LTP40*, *LTP41*, *LTP42*, *LTP48*, *LTP49*, *LTP53*, *LTP66*, *LTP67*, and *LTP75*—maintained consistent protein lengths across all diploid cotton species. While five genes—*LTP24*, *LTP52*, *LTP56*, *LTP70*, and *LTP81*—were absent in some diploid cotton species, the protein lengths in the remaining samples remained consistent. In contrast, considerable variability was observed in the protein lengths of other LTP family members.

To systematically investigate the evolutionary relationships of *LTP* genes in cotton and *Arabidopsis thaliana*, 107 *LTP* genes from cotton and 23 from *Arabidopsis thaliana* were analyzed together in this study, and a phylogenetic tree was constructed (**Figure 1D**). Based on the clustering results of the evolutionary tree, these *LTP* genes were classified into seven subgroups. Among the subgroups, LTP-f was the largest, containing 45 LTP genes, followed by LTP-g, LTP-a, LTP-e, and LTP-b, which contained 41, 37, 22, and 19 *LTP* genes, respectively. The LTP-d subgroup was the smallest, with only 17 LTP genes. Notably, no *LTP* genes from *A. thaliana* were detected in the LTP-c subgroup. In terms of overall homology, cotton *LTPs* presented high homology with those in *Arabidopsis thaliana*. In conjunction with the results from the previously described PAV analysis, three types of gene families—core genes, variable genes, and specific genes—were found in each subgroup. This suggests that the *LTP* family may not have been in a stable state throughout the evolutionary process but instead underwent dynamic changes.

### 3.2 Evolution of *LTP* in diploid cotton species

**Figure 2:**
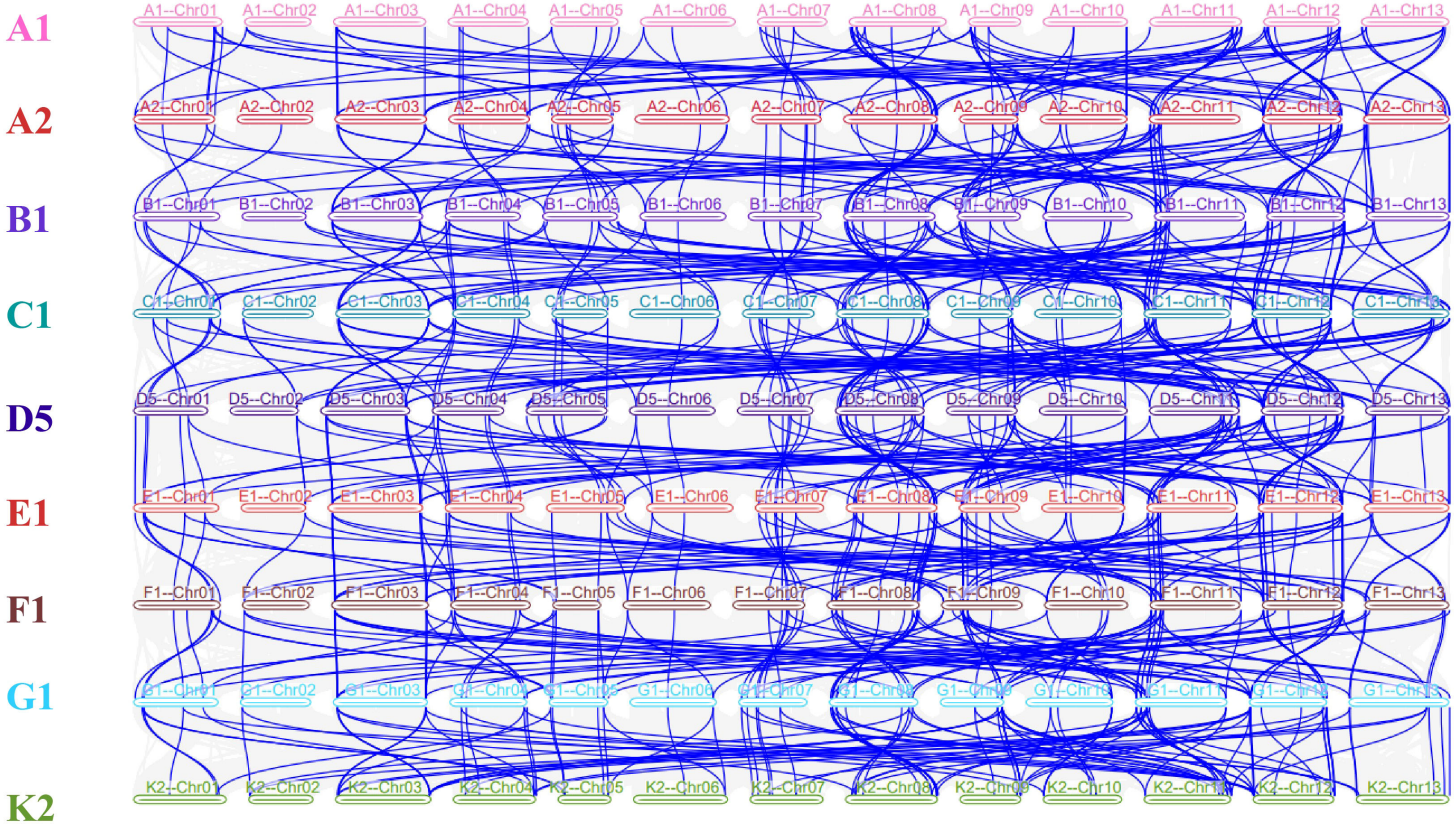
Colinearity analysis plot of LTP genes across nine diploid cotton genomes.

To explore the evolution of *LTPs* in diploid cotton, collinearity analysis was conducted across the genomes of nine diploid species. As depicted in **Figure 2**, numerous homologous *LTP* gene pairs are observed on the chromosomes of various diploid cotton species. For instance, the *LTP genes* in groups A1, B1, and C1 are found on similar chromosomes across different diploid cotton species, indicating that these genes likely existed in a common ancestor and were preserved during species differentiation without significant gene loss or rearrangement. However, exceptions exist, such as genes in the D5-E1-F1-G1-K2 group, which do not exhibit a clear collinear pattern. This may be attributed to increased recombination events or mutations, potentially caused by unknown factors.

**Figure 3:**
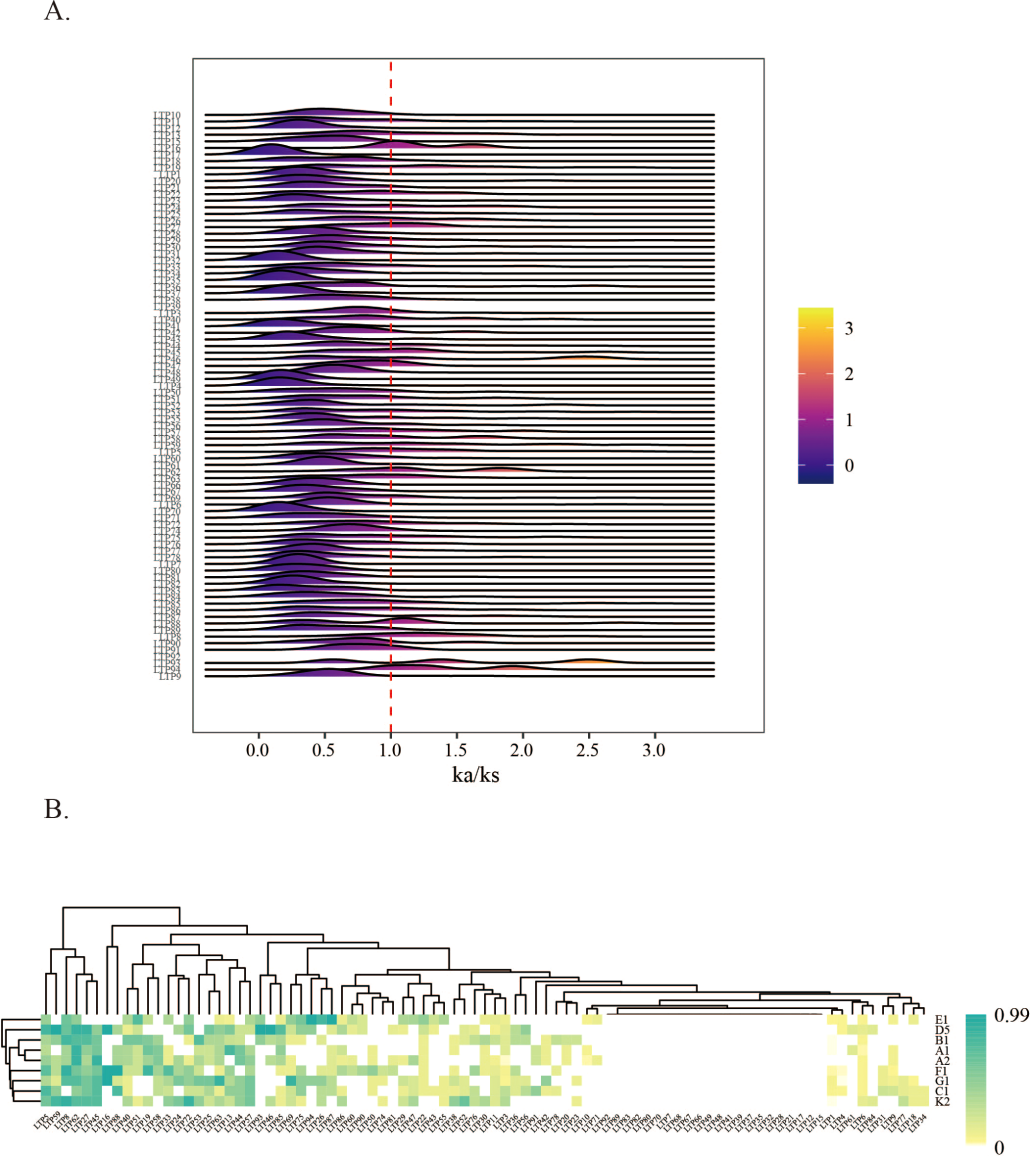
LTP gene Ka/Ks levels in diploid cotton species. **(A)** Distribution of Ka/Ks values of LTP genes across nine diploid cotton species. **(B)** Heatmap showing the frequency of occurrence of each LTP gene in different diploid cotton species with Ka/Ks > 1.

Ka/Ks value analysis reveals differences in selection pressures exerted on gene family members across cotton species during their evolutionary history. To investigate the selection pressure on *LTP* genes in diploid cotton species, Ka/Ks values for each *LTP* gene were calculated on the basis of *LTP* gene sequences from the genomes of nine cotton species. Since some genes were present in only two cotton species, only one Ka/Ks value could be obtained, while another 19 genes could not be determined, as they were unique to a specific cotton species. Thus, Ka/Ks values were obtained for 87 *LTP* genes, and their distribution among the nine cotton species is illustrated in **Figure 3A**. The findings showed that the majority of *LTP* genes had Ka/Ks values that peaked between 0 and 1, with peak positions differing for each gene. Additionally, the peak Ka/Ks values for the *LTP16*, *LTP8*, and *LTP27* genes exceeded 1. Specifically, the *LTP46* and *LTP93* genes presented the highest Ka/Ks values, ranging from 2.4 to 2.6. About 20% of the *LTP* genes were found to have Ka/Ks values exceeding 1, suggesting that they were subject to positive selection in diploid cotton species. Conversely, Ka/Ks values below 1 were observed for some *LTP* genes, suggesting that these genes experienced purifying selection.

A heatmap was subsequently constructed to display the frequency of *LTP* genes across different diploid cotton species (**Figure 3B**). The analysis revealed that, among the nine cotton species genomes, the proportion of Ka/Ks values greater than 1 was greater for the *LTP5*, *LTP59*, *LTP8*, *LTP62*, *LTP27*, and *LTP45* genes, indicating that these genes were subjected to selective pressure during cotton domestication. Moreover, a correlation between Ka/Ks values and gene families showed that all members of the LTP-b family had Ka/Ks values of less than or equal to 1, suggesting that this subfamily experienced purifying selection and displayed comparatively conserved traits throughout evolution. These evolutionary differences among species underscore the significance of investigating interspecies variations to reveal how selective pressures have shaped LTP gene evolution in cotton.

### 3.3 Structural variation affects *LTP* gene expression and structure in diploid cotton species

**Figure 4:**
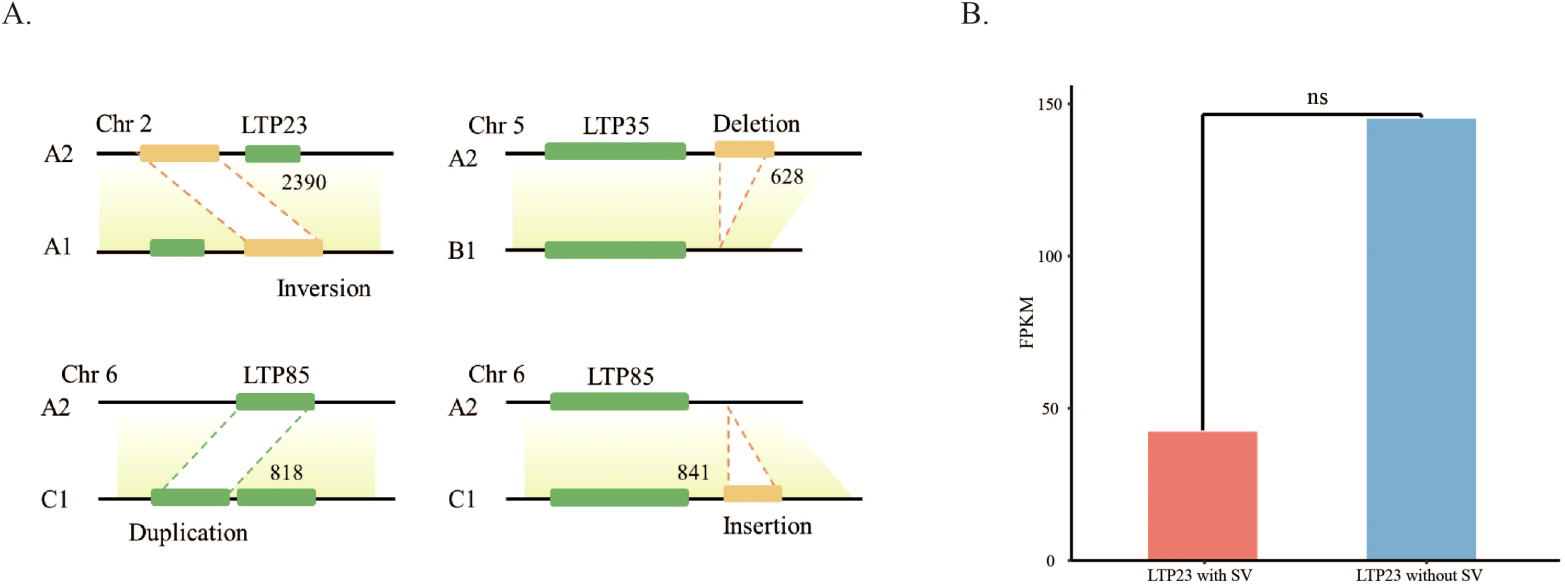
Effect of SV on genes. **(A)** Displays SV inversions, deletions, duplications, or insertions 2 kb upstream or downstream of the LTP gene. **(B)** Structural variations did not significantly impact the expression of LTP genes.

In the study by Wang et al (Wang et al., 2022), the genomes of eight high-quality diploid cotton species were analyzed in comparison to the reference genome (A2), resulting in the successful identification of numerous structural variants. Further analysis was conducted on the basis of the data mentioned above, revealing that a total of 2,740 SVs overlapped with the 2-kb upstream and downstream regions of 80 genes in the *LTP* gene family. Specifically, regarding the distribution across different gene regions, the downstream region contained 669 SVs, the exon region contained 260 SVs, the intron region contained 731 SVs, the RNA splicing-related region contained 4 SVs, the upstream region contained 1,004 SVs, the 3’ UTR contained 51 SVs, and the 5’ UTR contained 21 SVs. Compared with the reference genome, these structural variants were classified into five characteristic types: deletion (DEL), insertion (INS), duplication (DUP), inversion (INV), and translocation (TRA) (**Figure 4A**).

To examine how structural variation (SV) influences *LTP* gene expression, a series of analyses were carried out. To ensure the reliability of the results, genes with fewer than two samples were excluded before statistical analysis. The expression levels of genes that overlapped with SV and those that did not were then subjected to T tests and Wilcoxon tests, respectively. The analysis indicated a trend in some genes, with the *LTP23* gene showing the smallest p value (0.06) among the genes eligible for calculation (**Figure 4B**). While a potential effect of structural variation on LTP gene expression is suggested, the p-value failed to meet the statistical significance threshold (0.05). Thus, no significant correlation between structural variation and the expression of the LTP23 gene can be confirmed.

**Figure 5:**
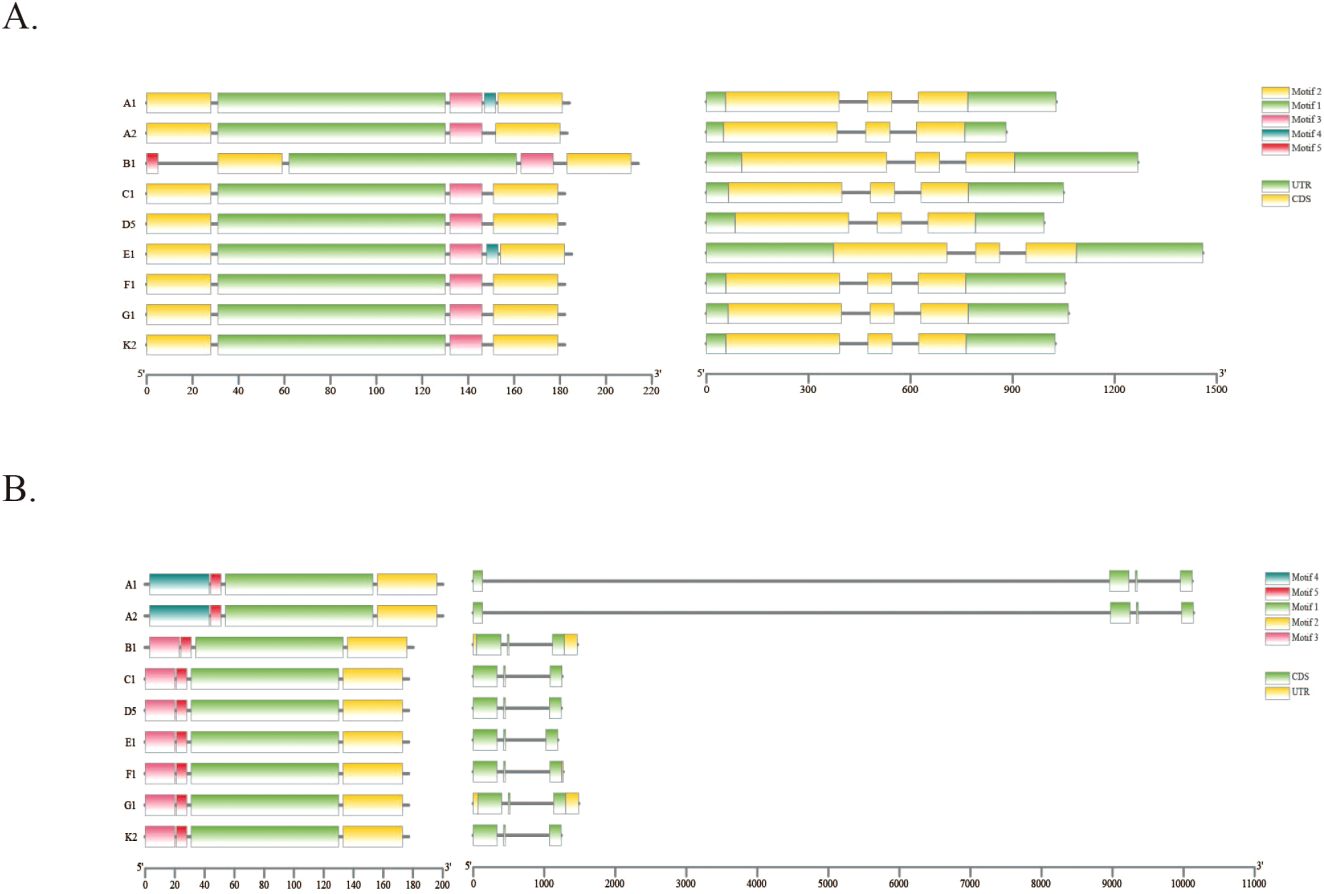
Gene structure of LTP in the genomes of nine diploid cotton species. **(A)** Gene structure of LTP35. **(B)** Gene structure of LTP85.

To investigate the impact of structural variations (SVs) on *LTP* gene family structure in diploid cotton species, we employed TBtools to analyze *LTP* gene structure across nine diploid cotton species. The results, shown in **Figure 5**, indicate that the structure of certain *LTP* genes overlapping with SVs has been altered. For example, the structural elements, coding sequences, and noncoding regions of *LTP35* exhibited corresponding changes across different cotton species. Specifically, motif 5 was added to the B1 genome, and motif 4 appeared in the A1 and E1 genomes; additionally, the CDS and UTR regions were modified. These structural alterations point to gene elongation in the LTP35 gene, which may result in functional alterations like pseudogenization or the creation of novel activities. Furthermore, the *LTP85* gene exhibited variable structural changes in the A1 and A2 genomes, as evidenced by a significant increase in intronic regions and the emergence of new motifs. Collectively, these results indicate that SVs have altered the structure of *LTP* genes.

### 3.4 SV leads to atypical *LTP* genes in nine diploid cotton species

**Figure 6:**
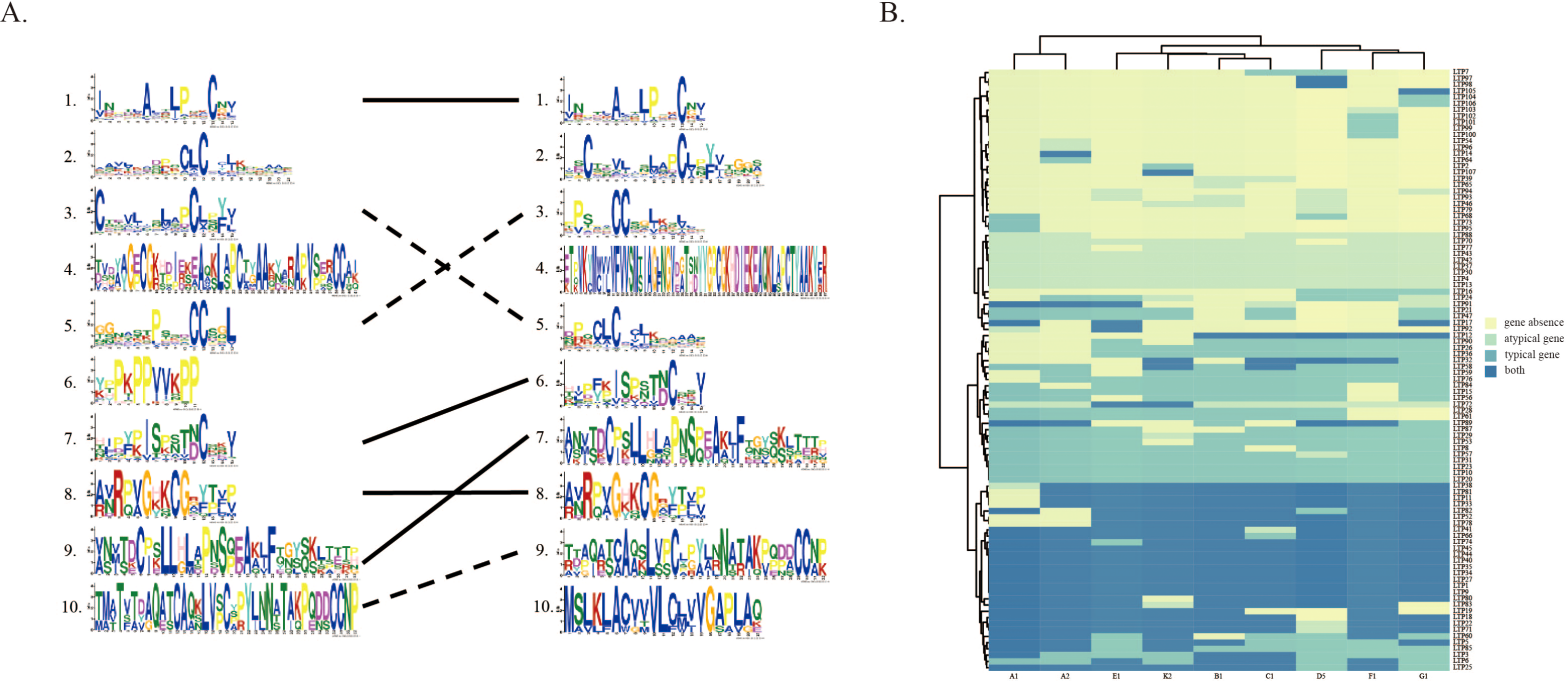
Structural variants (SVs) were found to influence the conserved domains of LTPs. **(A)** The left and right sides show the Weblogos of the reference genomes A2 and A1, respectively. The Weblogos connected by solid lines indicate correspondence, while those connected by dashed lines suggest similarity with partial changes. **(B)** Heatmaps illustrating the standard or anomalous traits of each LTP gene across many cultivars. “Both” signifies the existence of both conventional and unconventional genes.

Structural variations (SVs) are crucial factors that induce alterations in the spatial folding of proteins. To assess the impact of structural variants on conserved domains of *LTPs* in diploid cotton genomes, we focused on the A1 genome, which harbors the largest number of *LTP* genes overlapping with structural variants. The amino acid sequences of its LTPs were uploaded into the MEME tool and compared with those of the reference genome A2. The analysis revealed that of the 10 *LTP* gene sequences in the A1 genome, 4 were aligned with the reference genome A2. In three of these genes, the sequence of the conserved structural domains was modified, while the domains of the other three genes could not be accurately aligned with those in the reference genome A2 (**Figure 6A**). Further analysis of the amino acid changes within these 10 structural domains revealed that the structural variants influenced the conserved domains of various *LTP* genes.

Traditional gene family analysis methods typically exclude genes with changes in conserved structural domains from family identification, which often results in these genes being overlooked in subsequent functional studies. To explore the relevant aspects fully, we performed statistical analyses and displayed the results for atypical genes (lacking both PF14368 and PF00234 structural domains) across each variety (**Figure 6B**). The statistical analysis revealed that certain genes exhibited more complex classifications across different samples. For example, genes such as *LTP38*, *LTP81*, *LTP11*, *LTP33*, *LTP82*, and *LTP52* were classified as both typical and atypical genes in most samples. In contrast, genes such as *LTP88*, *LTP70*, *LTP72*, *LTP77*, *LTP43*, *LTP42*, *LTP37*, *LTP30*, *LTP4*, and *LTP13* were classified as atypical genes in many samples. Notably, these atypical genes are frequently disregarded in conventional gene family analysis methods that depend on individual genome references. The findings indicate that structural variants likely influence the conserved structural domains of LTP, resulting in the proliferation of numerous atypical genes across the nine diploid cotton species.

### 3.5 *Cis*-acting element analysis of promoters

**Figure 7:**
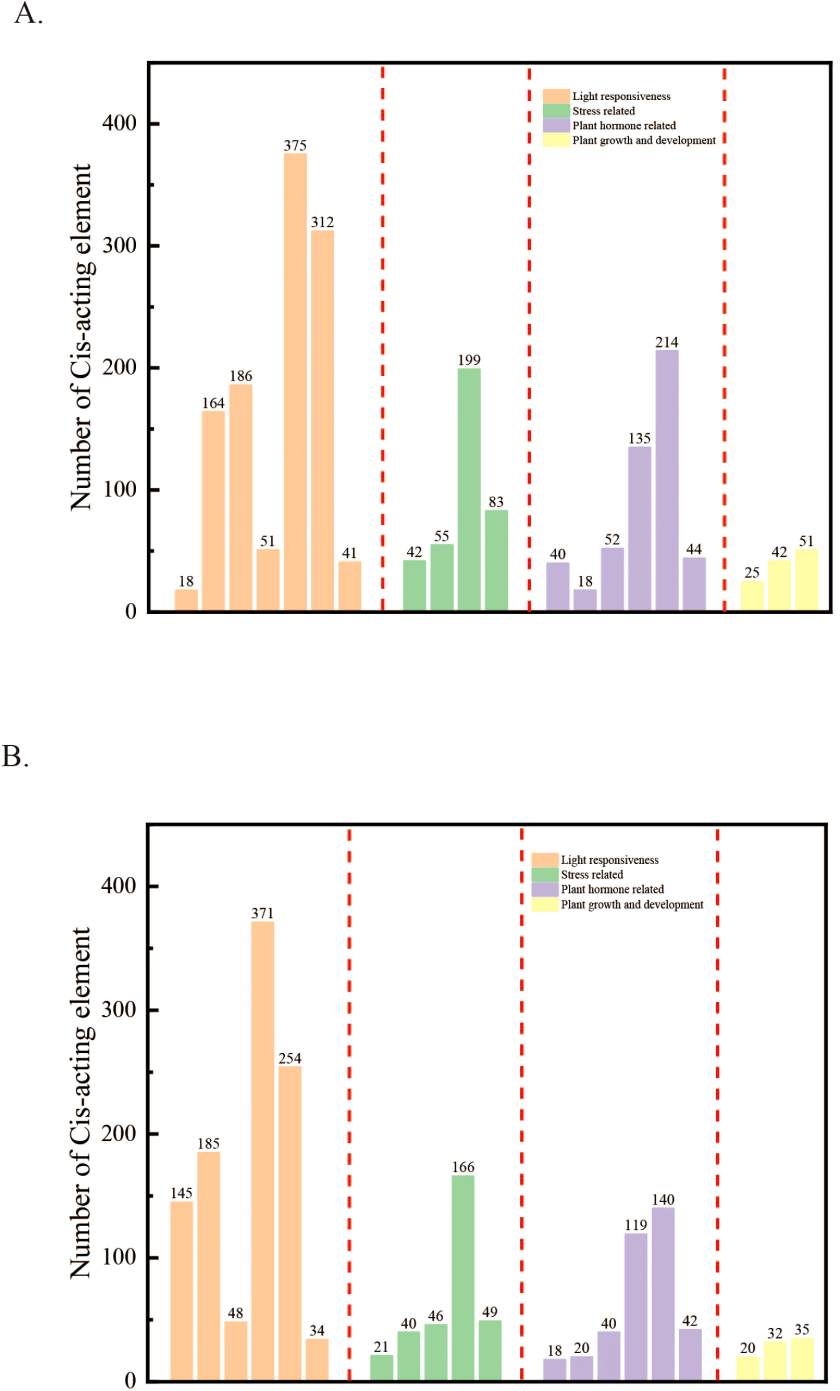
Statistical examination of the quantity of cis-acting elements for A2 and A1. **(A)** Statistics on the quantity of cis-acting elements for A2. **(B)** Statistics regarding the quantity of cis-acting elements for A1.

To thoroughly investigate the effect of structural variation on the composition of *cis*-acting elements, the A1 genome, which contains the greatest number of structural variations overlapping with the reference genome, was chosen as the focus of this study. The promoter region of this genome was examined for *cis*-acting elements, and the top 20 were chosen for statistical analysis and mapping (**Figure 7**). The findings indicated substantial disparities in the quantity of cis-acting elements associated with light and stress responses between the reference genomes A2 and A1. The A2 genome comprised seven *cis*-acting elements associated with light response and four associated with stress response, while the A1 genome included six light-response elements and five stress-response elements. Further comparison revealed that, in addition to the number of light- and stress-responsive *cis*-acting elements, the number of phytohormone-responsive elements also varied considerably between the A1 and A2 genomes. These differences suggest that structural variation can alter the composition of cis-acting elements in the promoter regions of *LTP* genes across different samples. Since *cis*-acting elements play a crucial role in regulating gene expression, alterations in their composition resulting from structural variation may affect important physiological processes, such as photosynthesis and plant stress responses.

### 3.6 Analysis of the spatiotemporal expression patterns of the *LTP* gene and construction of its transcription factor regulatory network

**Figure 8:**
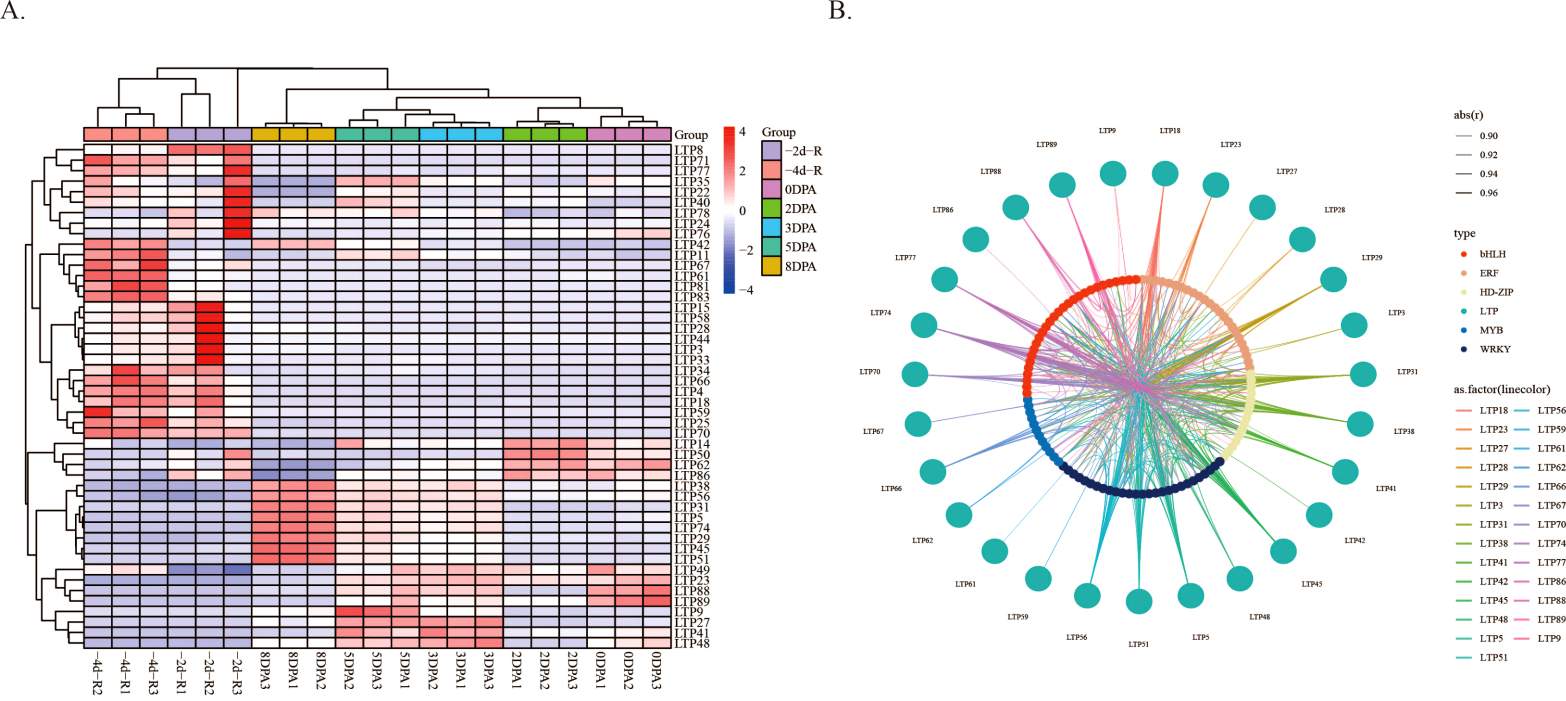
Differential expression heatmap and Coexpression network map for LTP. **(A)** Heatmap showing the differential expression of LTP genes at various time points. **(B)** Coexpression network between LTP genes and transcription factors.

To investigate the mechanism of *LTP* gene action during cotton fiber development, heatmaps of differential expression were generated for cotton ovules and roots at different developmental stages. Analysis of the heatmaps revealed a more distinct spatiotemporal stratification pattern (**Figure 8A**). Different *LTP* genes presented differential expression patterns across various tissues and developmental stages of cotton. Genes such as *LTP8*, *LTP71*, *LTP77*, *LTP67*, *LTP61*, *LTP81*, and *LTP83* presented tissue-specific expression and were detected exclusively in the roots at -2DPA and -4DPA. In contrast, *LTP38*, *LTP56*, *LTP31*, *LTP5*, and *LTP74* presented developmental stage-specific expression, which was observed only in cotton ovules from 0DPA to 8DPA. Additionally, genes such as *LTP35*, *LTP22*, *LTP40*, *LTP78*, *LTP14*, and *LTP50* exhibited dual expression characteristics, with expression detected in both roots and cotton ovules. The regulatory network of *LTP* genes was examined by calculating Pearson correlation coefficients between cotton transcription factors and differentially expressed *LTP* genes (**Figure 8B**). The analysis revealed that members of the WRKY, bHLH, and ERF transcription factor families were significantly correlated with the expression of *LTP* genes in diploid cotton species, implying their role in regulating the transcription of *LTP* genes.

## 4 Discussion

Feng et al. identified 104 *LTP* genes in *Gossypium raimondii* and *Gossypium arboreum*. After manual screening for cysteine residues, signal peptides, and amino acid lengths, 47 *LTP* genes were identified in Raymond cotton and 51 in Asian cotton, reducing the gene count by nearly half (Li et al., 2016). In this study, *LTP* genes from diploid cotton species, including Raymond cotton and Asian cotton, were recharacterized via high-quality pangenomic data from Wang et al. Preliminary screening identified 118 *LTP* genes in Raymond cotton and 105 in Asian cotton. Gene family identification typically filters out genes in HMMSEARCH and SMART because of incomplete conserved structural domains. Therefore, a slightly more relaxed criterion was adopted for the identification of LTP genes. Specifically, HMMER searches were performed using the Pfam domains PF14368 and PF00234, and the resulting sequences were filtered based on both E-value and alignment coverage. Genes showing ≥90% similarity to the reference domains were classified as typical LTP genes, whereas those exhibiting approximately 80% similarity, partial structural deletions, or amino acid sequence lengths less than 90% of those of typical LTP genes were designated as atypical LTP genes. Subsequently, both groups of genes were combined and further validated using the SMART and CDD databases to minimize false positives and ensure that potential LTP members, including atypical genes, were not overlooked. Ultimately, 90 *LTP* genes were identified in Raymond cotton and 82 in Asian cotton, which exceeded the findings of Feng et al.

Diploid cotton species exhibit significant conservation in chromosome architecture, gene arrangement (synteny), and homologous gene associations (Hu et al., 2024; Wendel and Cronn, 2002). To investigate the evolutionary trajectories of the *LTP* family in diploid cotton species, we analyzed the gene order, retention of homologous relationships, and covariance between chromosomes on the basis of collinearity analysis of *LTP* genes. This analysis suggested that *LTP* genes are relatively conserved across different diploid cotton species. We subsequently analyzed the *LTP* family members for selection pressure. Approximately 20% of the *LTP* genes exhibited Ka/Ks values over 1, signifying that this subset of *LTP* genes underwent selection pressure in diploid cotton species. This finding further confirms the relative conservation of *LTP* family evolution. We then analyzed the evolutionary relationships within the *LTP* family and classified the core, variable, and specific genes among the 107 *LTP* genes identified via PAV analysis. In total, 45 core genes, 43 variable genes, and 19 specific genes were identified. Core genes are prevalent in diploid cotton species, whereas variable and specific genes are either absent or expanded in some cotton species. This ensured genome complementarity to some extent across different cotton species. This pattern of presence and absence may be linked to the evolutionary pathways of gene families in different species (Heineman et al., 2009). These findings highlight the importance of examining interspecies variations, as differences in gene retention, duplication, and loss across species can provide crucial insights into how LTP genes contribute to species-specific adaptation and evolution. Amino acid length and selection pressure analyses revealed that core genes, such as *LTP1*, *LTP3*, and *LTP6*, are highly conserved in diploid cotton species. Their amino acid lengths exhibit low variability, and Ka/Ks values of less than 1 suggest strong purifying selection. These findings imply that these genes play indispensable roles in the fundamental biological functions of cotton (Su et al., 2024; Wang et al., 2019). In contrast, variable genes such as *LTP8*, *LTP27*, and *LTP62* presented greater diversity in amino acid length and Ka/Ks values. Higher Ka/Ks values suggest that these genes may be subjected to positive selection, reflecting their potential functional differentiation in response to the adaptation of diploid cotton species to specific environments (Zhong et al., 2015; Zia et al., 2022).

We examined the influence of structural variation on the *LTP* family by characterizing the structural variation of its members across several genomes. The findings demonstrated that structural variations caused alterations in gene structure and structural domains, although exerted no influence on gene expression. A structural variation study of the *cis*-acting elements was performed. The research indicated that structural variants resulted in variations in the content of cis-acting regions between genome A1 and reference genome A2. These changes may further modify the physiological functions of *LTP* family members in these two diploid cotton species. In summary, structural variants can impact the cotton *LTP* family at multiple levels, including gene structure, structural domains, and *cis*-acting element composition, potentially altering their physiological activities. Structural variation (SV) is a prevalent type of genetic mutation that induces alterations in conserved structural domains, potentially resulting in the exclusion of certain gene family members during identification. In cotton genome research, SV induces changes in gene order and structure, resulting in alterations in gene sequence characteristics that complicate identification via conventional methods (Yang et al., 2019). In this context, the discovery of atypical genes provides new insights into the evolution of diploid cotton species. As previously noted, we identified several atypical genes, including *LTP4*, *LTP13*, and *LTP88*. A combined analysis of these genes revealed that *LTP13* presented greater variation in amino acid length, whereas the remaining atypical genes (e.g., *LTP4*) presented relatively conserved amino acid sequences with minimal variation. Subsequent study, along with selection pressure, indicated that *LTP13* and *LTP88* exhibited Ka/Ks values exceeding 1 in certain cotton species, implying that these genes may have experienced positive selection pressure. Subsequent differential expression analysis revealed that atypical genes such as *LTP4*, *LTP42*, and *LTP88* were expressed in roots or ovules, suggesting a potential role in the growth and development of cotton. The emergence of these genes may have conferred new functions or adaptive advantages to cotton, leading to their retention throughout the evolutionary process.

The *LTP* gene family contributes to the development of many organs and responses to stress in cotton. The *LTP6* gene is exclusively expressed in fiber cells, with expression levels peaking at key stages of fiber formation, and may have a role in fiber cell wall construction (Ma et al., 1997). *GhLTPG1*, a GPI-anchored lipid transfer protein, was found to be expressed in elongating cotton fibers and the outer seed coat of ovules, with significant enrichment (Deng et al., 2016). Moreover, members of the LTP gene family are expressed in roots. *GhLTP4* is expressed in the roots and fiber cells of cotton and is implicated in the physiological process of fiber cell elongation (Duan et al., 2023). Moreover, this gene is significantly induced under drought stress, and transgenic cotton plants overexpressing *GhLTP4* exhibit increased drought resistance (Zhang et al., 2022). *GhnsLTPsA10* expression increased in roots under Xanthomonas stress and significantly decreased in leaves under insect attack, influencing broad-spectrum resistance functions against disease and insects (Chen et al., 2021). This study analyzed the differential expression of LTP genes in cotton ovules and roots at various time points, revealing a distinct spatiotemporal stratification pattern categorized into three groups: genes expressed exclusively in ovules, genes expressed exclusively in roots, and genes expressed in both ovules and roots. On the basis of existing reports, we hypothesized that the genes expressed solely in ovules and those expressed in both ovules and roots may share similar molecular mechanisms with previously reported *LTP* family members and may play a role in regulating key biological events in cotton fiber development. Whether genes expressed solely in the root exhibit a stress response function, similar to that of the *GhLTP4* gene, requires further investigation. We further analyzed the differential expression heatmaps of the core and variable genes. The results revealed that 27 core genes were specifically expressed in ovules or roots at different times: 55.6% were expressed in ovules, 29.6% in roots, and 14.8% in both. Additionally, 18 variable genes were expressed in ovules or roots at different times: 66.7% were expressed in roots, 16.7% in ovules, and 16.6% in both. Data analysis revealed that the majority of the core genes were expressed in ovules, whereas most of the variable genes were expressed in roots. These findings offer valuable insights into the functional differentiation of the *LTP* gene family in various cotton tissues.

On the other hand, The expression of cotton LTP genes is regulated by various transcription factors, including MYB-like (Duan et al., 2024), bHLH-like (Duan et al., 2023), ethylene signaling pathway-related, and stress-related transcription factors (Duan et al., 2024). In this study, a coexpression network map analysis that, in diploid cotton species, LTP genes are regulated not only by WRKY, bHLH, and MYB transcription factors but also by ERF and HD-ZIP factors. These results expand our understanding of the complex transcriptional regulation of the LTP gene family and provide new insights into their potential upstream regulatory mechanisms. We acknowledge that the expression analysis in this study is based on transcriptomic data, and mRNA abundance does not always correspond to protein-level regulation. For instance, Peng et al. integrated transcriptomic and proteomic analyses and found that, under salt stress, only a subset of genes showed consistent changes at both mRNA and protein levels, while most proteins with strong differential expression lacked corresponding transcript variation (Peng et al., 2018). Similarly, Chen et al. reported that the correlation between mRNA and protein expression was not fully consistent during the early and middle stages of salt stress, reflecting complex post-transcriptional and translational regulation (Chen et al., 2021b). In addition, Zhang et al. compared drought-tolerant and drought-sensitive cotton varieties and found that proteins associated with antioxidant activity, transport, and metabolism were significantly upregulated in the drought-tolerant variety, while their corresponding transcripts did not fully align (Zhang et al., 2016). These findings support the reliability of transcriptome-based co-expression networks as predictive tools but also underscore the necessity of future validation of representative LTP genes through proteomic, metabolic, and functional studies.

**Figure 9:**
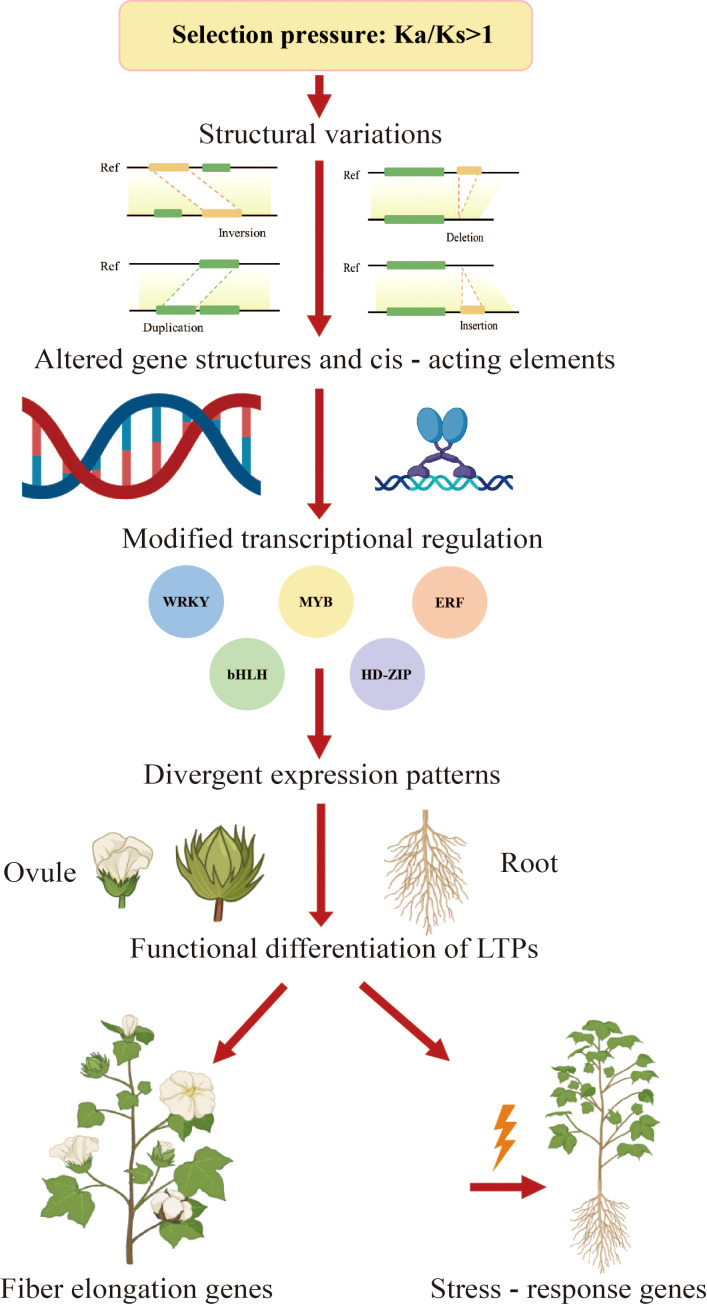
Schematic model of the evolutionary and functional diversification of the LTP gene family in diploid cotton species. Selection pressure drives structural variations that alter cis-regulatory elements and transcription factor interactions, leading to expression divergence and functional specialization of LTP genes. Representative genes (e.g., LTP6, LTP35, LTP4, and LTP88) are involved in fiber development and stress responses.

Although this study provides a comprehensive genomic and transcriptomic framework for understanding LTP gene regulation, functional verification remains an important next step. Future studies will focus on experimental validation, including RT-qPCR expression profiling and genetic manipulation (such as overexpression or knockout analysis), to confirm the regulatory roles and biological functions of key LTP genes identified here. To provide a visual summary of these findings, a schematic model was constructed to illustrate how selection pressure and structural variations have shaped transcriptional regulation, expression divergence, and functional differentiation of the LTP gene family in diploid cotton species (**Figure 9**).

## 5 Conclusion

This paper offers a comprehensive characterization of the *LTP* gene family in diploid cotton species, grounded in a high-quality cotton pan-genome. The findings indicated that the *LTP* family exhibits generally preserved traits throughout the evolution of diploid cotton species and highlighted the impact of structural variation on the quantity of *LTP* family members, gene architecture, conserved structural domains, and cis-acting elements. Subsequent analysis indicated that this gene family is both conserved and variable in the evolution of diploid cotton species: the conservation of core genes guarantees the stability of fundamental biological functions, while the diversity of variable and specific genes may enhance cotton’s adaptability to various environments. This study found unusual genes with biological activities. Building on these findings, subsequent research can investigate the precise roles of these genes in cotton fiber growth and environmental adaptability, elucidating the fundamental mechanisms driving the evolution of diploid cotton species. Future studies should also pay more attention to interspecies variations to better understand species-specific evolutionary trajectories of the LTP gene family.

## Data Availability

The original contributions presented in the study are publicly available. This data can be found here: NCBI Sequence Read Archive (SRA), accession number PRJNA1368280, https://www.ncbi.nlm.nih.gov/bioproject/PRJNA1368280/.
